# Smokeless Tobacco in Uganda: Perceptions among Tobacco Control Stakeholders

**DOI:** 10.3390/ijerph19063398

**Published:** 2022-03-14

**Authors:** Denis Male, Shirley Kansabe, Hafsa Lukwata, Alexander Rubanga, Kamran Siddiqi, Linda Bauld, Ann McNeill, Fiona Dobbie

**Affiliations:** 1 Department of Food Technology and Nutrition, School of Food Technology Nutrition and Bioengineering, College of Agricultural and Environmental Sciences, Makerere University, Kampala P.O. Box 7062, Uganda; kansabe.shirley@gmail.com; 2 Mental Health Division, Ministry of Health, Kampala P.O. Box 7272, Uganda; hafluk@yahoo.com; 3 International Affairs Unit, Uganda Revenue Authority, Kampala P.O. Box 7279, Uganda; arubanga@ura.go.ug; 4 Department of Health Sciences, University of York, Seebohm Rowntree Building, Heslington, York YO10 5DD, UK; kamran.siddiqi@york.ac.uk; 5 Usher Institute, College of Medicine and Veterinary Medicine, University of Edinburgh, Edinburgh EH8 9AG, UK; linda.bauld@ed.ac.uk; 6 Department of Addictions, Institute of Psychiatry, Psychology & Neuroscience, King’s College London, London SE5 8BB, UK; ann.mcneill@kcl.ac.uk

**Keywords:** smokeless tobacco, law enforcement, stakeholder perception, illicit trade, comprehensive ban

## Abstract

The use and sale of smokeless tobacco (SLT) is prohibited in Uganda under the Tobacco Control Act (TCA), 2015. Nonetheless, SLT products remain available, and there are limited and inconsistent data on SLT users. Additionally, the perceptions of tobacco control stakeholders on SLT are unknown, making it difficult to determine barriers to enforcing the ban. This study examined perceptions of tobacco control stakeholders regarding SLT in Uganda. Qualitative semi-structured interviews were conducted with stakeholders who were purposively selected from ministries, semi-autonomous government agencies and Civil Society Organizations. Interviews explored knowledge, attitudes, perceptions of SLT appeal, and user demographics. Data were analysed using Nvivo V.12 software. Participants demonstrated a general lack of awareness of SLT product types and the extent of their use. They believed SLT use was increasing among females and minors and was as harmful to health and the economy as smoking. SLT products were thought to be cheaper than cigarettes and to appeal to minors. Discreet use was thought to help users overcome the cultural aversion towards tobacco use among women and youth in Uganda. There is an urgent need to strengthen the implementation of the SLT ban whilst also increasing efforts to reduce tobacco smoking.

## 1. Introduction

Smokeless tobacco (SLT) products contain toxicants that pose significant health risks to users [1]. SLT covers a heterogenous range of products that have no proven safe level, which means that any consumption is harmful to health, albeit lower in comparison with combustible cigarettes [1,2].

The 2015 Uganda Tobacco Control Act (TCA) comprehensively banned the use of and trade in SLT. According to Uganda’s law, SLT means “products entirely or partly made of the leaf tobacco as raw material which are manufactured to be used for chewing, sucking, sniffing or any other means of oral consumption”. As part of the implementation mechanism, the law authorized environmental inspectors, customs, standards, public health officers, and any person whose function is to keep law and order, to enforce the TCA. In addition, the law provides for the Minister of Health to appoint authorized persons or groups including members of Civil Society Organizations to enforce the Act. Authorized enforcement officers have the power to search premises and confiscate and destroy any products that do not comply with provisions of the Act, thus enforcing the comprehensive ban of SLT. The law further prescribes an individual penalty of 24 currency points (Approx. USD 130) or imprisonment for not less than one year or both and 1000 currency points (approx. USD 5400) for corporations [3].

Despite the comprehensive ban, SLT products are widely available in Uganda. There are several SLT products sold in Uganda, and they differ considerably in both processing and how they are consumed. The products include modern-packaged industrially manufactured SLTs, primarily imported from Asia, and unstandardized products produced by local artisanal processors. However, data on the prevalence of SLT use are limited and inconsistent; some indicate a higher prevalence among women [4,5] while others show greater use among men [6]. Of greatest concern is that the highest SLT prevalence is among adolescents aged 13–15 years, accounting for 6.5% (7.1% and 6.0% in boys and girls, respectively) [7]. A key challenge to the implementation of the comprehensive ban is the limited information on knowledge and perceptions of tobacco control enforcers on SLT products sold in Uganda. This makes the readiness of government agencies to enforce the tobacco control laws relating to these products difficult to ascertain or assure [unpublished TCCP stakeholder workshop on 24 October 2018]. A previous study conducted among Police Officers who enrolled in the national drug interdiction training course in the US found that the officers’ attitudes towards drugs had an impact on how aggressively they enforced the laws and the discretion they employed [8]. Therefore, the knowledge of key stakeholders with tobacco control remit in Uganda and their perceptions are important in identifying and addressing the illicit trade and use of SLT.

The aim of this study was to explore tobacco control stakeholders’ perceived level of awareness of SLT products and their associated health risk, promotion and marketing strategies of the tobacco industry, and perceptions of their appeal to users. These factors may influence the enforcers’ likelihood to actively pursue enforcement of the SLT ban in accordance with Uganda’s tobacco control law.

## 2. Materials and Methods

### 2.1. Sampling and Recruitment

We conducted 17 individual face-to-face and 3 online (web-conferencing) interviews with tobacco control stakeholders working in both Government and Civil Society Organizations in the Kampala and Busia Districts. Participants were purposively recruited due to their remit to support the implementation of the comprehensive ban on SLT or their involvement in tobacco-control-related work in Uganda. To ensure there was sample diversity, participants were interviewed from Civil Society Organizations (*n* = 5), Government Agencies (*n* = 7) and Ministries in Uganda (*n* = 8). Participants voluntarily took part in the study and were not reimbursed or compensated for their contribution to the study. Participants gave written informed consent before the interviews started.

### 2.2. Data Collection

Data were collected between August and October 2020. Semi-structured interview guides were used to explore perceptions of stakeholders on SLT use in Uganda. Participants were asked about their knowledge and attitudes towards SLT. The in-depth interviews included discussion of the content of SLT, the demographic characteristics of the users of SLT, the drivers of use of the products, as well as how the harms of SLT and cigarettes compare. The semi-structured design of the interview guide contained discussion points but also allowed for detailed probing and flexibility when appropriate. The topic guide was pretested and refined before being used on the participants. Interviews were audio recorded and, on average, lasted about 45 min, with a range of 30 to 60 min.

### 2.3. Data Analysis

The 20 interviews were transcribed verbatim, with inductive and abductive approaches being used to analyze the transcripts. An inductive ‘bottom up’ analytical approach, with its focus on describing patterns and associations, was well placed to answer the research questions of interest. Abductive approaches were then used to develop an understanding of these patterns and associations by focusing on the ‘insider’ views (i.e., the research participant) rather than imposing a ‘top down’ researcher perspective [9,10]. Using NVivo V.12 Plus, DM, FD, and SK coded three similar transcripts independently using the line-by-line open-coding approach. They then compared their coding and discussed and developed an initial coding frame which was updated throughout the subsequent coding. FD coded a further four transcripts, while SK and DM coded the rest. Regularly, we discussed the independent coding to ensure it captured new ideas. After coding, SK and DM independently reviewed all the coded data to compare and check emergent themes and sub-theme classifications.

## 3. Results

From our analysis, we identified several themes which summarized stakeholders’ perceptions of SLT. These centered on four key areas: SLT awareness and extent of use; perceptions of SLT harm; strategies used to promote SLT and target consumers; and finally, considerations regarding the appeal of SLT.

### 3.1. Limited Levels of Awareness of Smokeless Tobacco Products and Their Use

Participants reported a general lack of awareness of what SLT products were and how prevalent their use was. They further noted that there was limited evidence about SLT use in Uganda, which could result in SLT not being viewed as a priority for tobacco control. For example, participants from Non-Governmental Organizations (NGOs) working in tobacco control commented on their lack of understanding of both the extent of use SLT and the health harms it can cause:

*… I think for me, it’s a silent challenge, we don’t know about it, I am not even sure that the people in public health really are paying attention to it, so, I’ve clearly, I’ve seen, I don’t have statistics, I don’t know if somebody has done a study but what I’ve seen physically, and even speaking to young people as somebody interested, this is increasingly being used—Stakeholder, CSO.*

This general lack of awareness of SLT was also perceived to be applicable to users of SLT, with participants noting their concern that users of SLT were not aware of the dangers associated with the products:

*… People do not have information on the dangers of tobacco, the different types of tobacco, you know, everything that tobacco can cause. Especially the young people. They don’t know—Stakeholder, Government Ministry.*

There was also a view that SLT consumption was becoming increasingly gendered. Participants felt that SLT use was increasing among young females, including those who are still in school:

*… the demographics are shifting, they’re shifting heavily towards the female gender, but also, there’s an age aggregate to the young people. But also because of affordability, because there’s vulnerability in the gender—Stakeholder, CSO*.

*… the demographics are that there are more women, more young people, and school going and out of school who are using kuber [SLT product] than those that are using cigarettes—Stakeholder, CSO.*

Participants felt that SLT presented an opportunity for more females to use tobacco. These findings represent a shift from the general norms since, culturally, women in Uganda do not normally smoke. However, some older women in especially rural settings consume SLT in the form of unprocessed raw tobacco leaf. This view was echoed by policy makers working in the health sector, civil society, as well as the police. Their perception was that women were afraid to smoke in public and so would prefer to use the more concealable SLT products:

*… I think, it is known that females are not meant to smoke, they’re some…, okay those are cultural things, eh? It’s not okay for a woman to smoke and people have listened to that. So, the industry must have found ways to entice those who feel that smoking is not good to also continue to use tobacco eh? So, yes. I think it is more amongst women, it is more amongst women than for males because of that kind of fear. It can easily be disguised for anything—Stakeholder, Government Ministry.*

### 3.2. Smokeless Tobacco Was Thought to Be as Harmful and as Addictive as Smoked Tobacco

Perceptions of SLT harm were discussed in three ways. First, there was a strong belief among stakeholders that SLT is as harmful as smoked tobacco. This was based on the reasoning that both products are derived from the same source, i.e., the tobacco leaf. Participants were aware of the health consequences of consuming SLT and reported that SLT could cause intestinal ulcers, dizziness, tooth decay, impotence, heart disease, respiratory diseases, cancers, and several nervous system issues. That said, participants did not discuss different types of SLT and relative harms from varied products:

*… for as long as your raw material for the product is a tobacco leaf, and you use any of those products, then you are actually exposed to the whole cocktail of chemicals that come from the tobacco leaf—Stakeholder, Government Ministry.*

*… I wouldn’t say the effect is less, actually most of them have been associated with the stomach cancer and cancer of the mouth. So, they are as dangerous and life threatening as these ordinary tobacco products—Stakeholder, CSO.*

Next, was a belief among interviewees that SLT products were equally as addictive as cigarettes. Both Government and CSOs thought that the addictiveness of SLT was attributed to their nicotine content, as well as additives that tobacco companies use during industrial processing or manufacture of SLT products. There was also a perception that industrially processed SLT products could be more addictive than locally produced, minimally processed SLT products:

*… For as long as it is a tobacco product and has nicotine. Then the addiction will come because you’re taking in nicotine whether it’s through chewing or sniffing or smoking. So, the addictive nature of the products is the same because of the same ingredient which is nicotine. The nicotine will behave the same way through smokeless and through smoked—Stakeholder, Government Ministry.*

*… I know that the addiction, at the factory they put in some other chemicals that will help the nicotine go faster to the brain. But that doesn’t mean that you’re more addicted. Once you’re addicted, really, there’s no difference. To me, there’s no difference. I can say you get addicted faster with processed products. Because they’re putting some chemicals to activate that – Stakeholder, CSO.*

Participants also reported beliefs that addictiveness could vary amongst SLT products depending on whether the products were sniffed/inhaled or chewed. Furthermore, there was a view that inhaled SLT was absorbed faster and reached the brain more quickly than SLT products that were chewed:

*… The level of addiction depends. It depends on how this product is. Just like I said, the ones that are snuffed are very addictive and it’s very easy for someone to use it and get their high. But for the ones that are chewed require a lot – Stakeholder, Government Ministry.*

A final point in relation to perceptions of SLT harm was the potential for negative consequences on the economy, as treating SLT-related conditions contributes to the cost of healthcare. In addition, since SLT is illicit in Uganda, the government does not receive any tax revenues from the presence of the products on the market, thus exacerbating the economic deficit:

*… So, government, has an extra burden on whoever falls sick as a result of consuming or being exposed to kuber (SLT product) or smokeless tobacco products. And that burden is not even affordable. So, it affects health financing. It affects government priority budgeting or expenditure or even performance because even it affects the health—Stakeholder, CSO.*

### 3.3. Promotion of Smokeless Tobacco Products Targets Youth and Minors

There was a view from some participants that young people under the legal age of tobacco consumption (21 years old) were being deliberately targeted by SLT manufacturers and traders. Enforcers believed that the tobacco industry used SLT to target children and young people to expand their market base in Uganda. This related to the view from participants that SLT use was increasing among children and that underage tobacco users were becoming addicted and felt the need to continue using concealable forms of tobacco:

*… It [Tobacco Industry] is targeting a very fragile age, it is hooking them into addiction, it’s going to disorganize their future, we cannot keep quiet – Stakeholder, Government Ministry.*

One of the perceived reasons for this increase in consumption of SLT among young people centered on a belief that the tobacco industry has intentionally focused on product designs to appeal to this age group. Participants commented that the products were designed to resemble snacks that appeal to children and often used attractive coloring, food-like flavoring, and shapes:

*… it’s packaged in a manner, that makes it look like the products that the young people like. Because, if you see like the tin, uhm which has chocolate tobacco, the tin actually looks like a chocolate tin, the colors are chocolate colors but also the flavor, when you open – Stakeholder, CSO.*

Participants also commented that one of the marketing strategies used to promote SLT products was to highlight aspects that may appeal to school-aged children. For example, highlighting the products’ ability to keep children alert in class and to boost their brain capabilities:

*… according to what I heard then was that it…, it helps you to keep awake…you know, to keep alert, uhm…, to be active in class. And that they were told that after all it’s a mouth freshener – Stakeholder, Government Ministry.*

*… it’s because of the way it has been marketed – it is being taken in as an appetizer, as something that will help people to stay up awake for you know, as a brain booster…. So…, if you tell a child about boosting their brains, they will be interested. And this is something they will gladly take on as young people. So, this one is the one that is affecting its increased use. Of course, most young people want to get very high grades so they can do anything – Stakeholder, Government Ministry.*

### 3.4. Affordability and Discreetness Could Explain the Appeal of SLT among Women and Youths

Our final theme is that of appeal, which considers participant perceptions of why consumers would use SLT. We identified four dimensions to this theme. The first was affordability. As noted previously, SLT is banned according to Uganda’s Tobacco Control Act, 2015. Therefore, all SLT products sold are illicit and have not paid any tax. For this reason, such illicit tobacco is expected to be cheaper than taxed cigarettes. Participants from CSOs and primary enforcement agencies including the Ministry of Health and the Police believed that SLT products are made cheap to target young people whose incomes are often lower than that for adults. Additionally, most chewable tobacco products are sold in small, low-priced sachets that enable lower-income strata to access them:

*… So, in terms of pricing, the ones that are smuggled you find that people get them cheaper because they don’t incur the same costs as the ones which are sold formally—Semi-autonomous Government Agency.*

*… these small sachets things, they’re affordable, because they tend to break them into affordable quotas that they pick that – Stakeholder, CSO.*

Next was the discreet nature of SLT products, which were thought to appeal to the high-income demographic. This view was shared by participants from both government enforcement agencies and civil society:

*… I would say it is consumed majorly by the urban elites, and ah…, if you look at ah…, they’re also sold in supermarkets, what you would call the kuber [SLT product], and its consumption is majorly by the urban guys, the urban elites—Stakeholder, CSO.*

*… The upscale. The middle class. Yaa. I feel like they’re the ones who usually use them – the smokeless tobacco products…, the people…, the elite, let’s just call them maybe the elite. Yaa. They’re the ones who actually don’t want to even be seen holding cigarettes, so they’ll smoke it in other forms—Semi-autonomous Government Agency.*

These stakeholder views suggest that SLT products appeal to both high-income and low-income tobacco users, especially in urban settings. The key selling points highlighted include their lower prices that make them affordable to the young people, as well as their ease to disguise or consume discreetly.

Next was the ability to consume SLT more discreetly than cigarettes. In Uganda, there is a general increasing stigma towards smoking in public and tobacco users are aware of the trend. Therefore, addicted tobacco users may find SLT products convenient to use since they are easy to disguise or use discretely. In addition, SLT can be consumed regardless of presence of other non-tobacco users:

*… So, I walk into a shop, pick it, put into my bag, so, I find it much easier to consume it, and…, I can be in a home, and nobody ever notices that I am consuming a tobacco product—Stakeholder, CSO.*

*… Because of the comfort people find in their usage, the ease with which they can move from one place to another, and when the person consumes it, he’s never detected—Stakeholder, CSO.*

The final point relevant to the appeal of SLT relates back to the gendered nature of consumption discussed previously. Modern industrially produced SLT products overcome the cultural aversion towards smoking among women. They are easier to conceal and disguise during use. In addition, their aesthetic attributes make them more appealing than the raw leaves commonly consumed by the older women. Some respondents from non-governmental agencies felt that such processed products appeal to and encourage tobacco use among younger females:

*… The females are not expected to smoke in public. So, they would rather…. They would smoke in hiding but use a little bit more of the smokeless compared to the males. Because the males would easily freely smoke—Stakeholder, Government Ministry.*

*… Now, that is flavored, that is packaged more creatively, uhm, it doesn’t look as crude, and as unattractive as the other traditional raw one. Uhm, yaa, so, that is attractive to the young people—Stakeholder, CSO.*

## 4. Discussion

Current evidence regarding the views of professionals working in tobacco control about SLT in low- and middle-income countries such as Uganda is limited. Existing peer-reviewed publications are comparative studies that focus on users, especially smokers [11,12,13]. To the best of our knowledge, this is the first study that specifically sought the views of tobacco control enforcement officers and other key stakeholders in Uganda. The results presented illustrated the favorable illicit trade environment characterized by limited knowledge among stakeholders on SLT and the prevalence of use in Uganda. In addition, the perceived discreetness, affordability, and gendered nature of SLT enabled its clandestine use by women who, culturally, would not be expected to smoke. Participants were overwhelmingly uninformed about the relative harm of SLT and combustible tobacco products; they believed that SLT is as harmful as cigarettes and felt that various product features and promotional strategies employed by the tobacco industry to lure minors towards SLT use were effective.

The perceived knowledge of SLT among government and non-government TC stakeholders in Uganda was generally low. This could be attributed to their novelty as well as the fact that SLT trade is an “underground business” or illicit. Participants also believed that the use of SLT is increasing among women and girls in Uganda. They argued that because in Uganda the prevailing norm is for females not to smoke, SLT may provide a convenient means for women and girls to use tobacco. The respondents also felt that the convenience is likely to influence more females to use SLT than males. Our findings were consistent with reported trends that indicated that the use of non-cigarette forms of tobacco was increasing among women [14,15,16,17]. Likewise, in Burkina Faso, it was observed that women prefer to use chewable smokeless tobacco, an act which is socially permissive [18]. While the trends of SLT use in Uganda mirror those in South and Southeast Asian nations, the cultural alignment and acceptance of SLT use for women is different and warrants deeper research.

Our discussions revealed that some tobacco control stakeholders had inaccurate information on the relative harms of SLT. They believed that SLT products were as harmful as smoked tobacco. This misconception echoed an earlier report from South Africa that indicated that participants thought the effects of SLT were comparable to those caused by cigarettes [13].

Participants also felt that SLT was either as addictive or more addictive than conventional cigarettes. Evidence from past studies indicates that perceived addictiveness varies with several factors. For instance, older people (over 65 years) were shown to be less likely than the youths (18–34 years) to believe that non-cigarette forms of tobacco including SLT, hookah, and cigars, were addictive [19]. In some studies, perceived addiction was strongly related to duration, frequency of use per day, hours of exposure per day [20], and prior exposure to information on SLT health effects [19]. Using smokers in a clinical trial setting, Hatsukami et al., 2016 showed that perceived health risks of snus (SLT product) were more accurate if the respondents consumed the product [21]. Our discussions sought opinions of stakeholders involved in law enforcement regardless of SLT use status. While lack of direct user-experience could have been a limitation, opinions of individuals involved in tobacco control law enforcement offer a unique context on drivers of law implementation. Misperceptions of relative harms and addictiveness of SLT and smoked tobacco could also result in stakeholders not focusing adequately on continuing, or indeed increasing, their efforts to reduce smoking, including greater regulations on smoked tobacco.

Participants from NGOs and some government ministries felt that the tobacco industry employed numerous marketing tactics to lure minors into using SLT. They cited cheap smuggled tobacco, packaging in small quotas, and in some instances, dispensing SLT into unstandardized packaging materials, making it difficult for authorities to detect. Participants felt that, as a result, SLT use is increasing among children and youth in Uganda. SLT use among minors appears to be increasing in several other African countries. Agaku et al., 2014 described SLT use among youth and minors (9–19 years) in Africa as an emerging epidemic [22]. Several other studies concurred that the colors, packaging, size, as well as the marketing of smokeless tobacco products by the tobacco industry drove its use among the youth and children [23,24,25]. Similar to advertising, it was thus noted that packaging characteristics of SLT could generate product preferences and appeal [26].

The perceived appeal of SLT to users in Uganda is discussed under three sub-themes: perceived affordability, their discreetness, and the gendered nature of SLT use as were reported by participants. First, on affordability, TC enforcers believed that non-payment of tax (SLT is illicit) and subsequent low cost of SLT could favor its increased consumption. Hypothetically, taxation could reduce the demand for SLT [27,28] (if SLT were legal products), although the heterogeneous nature of the products would be an impediment to taxation as a tool to decrease its use [27]. Nevertheless, the current comprehensive ban on SLT represents a best practice as recommended at the sixth Conference of Parties meeting in 2014 [29]. Therefore, there is need to strengthen the enforcement of the SLT ban to restrict illicit trade. Our study also revealed a popular view among law enforcement stakeholders that SLT products were easy to conceal and therefore convenient to use in public places where cigarette smoking would be abhorrent. This finding is consistent with observations in several previous studies [30,31,32]. However, some studies argue that consuming SLT is not that easy and requires some skill. One needs to learn how to ‘pack the lip’ and contain the tobacco between the lip and gum for extended periods until the nicotine and flavors are exhausted [32].

Another notable trend was the cultural shift where women and girls were thought to take up SLT use. One reason for this could be the ease to use SLT discreetly as discussed above, but also, there were sentiments that suggest product novelty as a possible driver of first-time use. Similar sentiments were documented in the US where participants generally reported positive perceptions of SLT because of their novelty. They used terms such as “hip”, “cute”, “in thing”, and “sleek”, among others, to describe their packaging as being aesthetically attractive [30]. Thus, there is a need to study the socio-cultural environment of the female SLT users in Uganda to elucidate the role it plays in influencing maiden SLT use.

## 5. Conclusions

The stakeholders’ perceived level of awareness of the dangers and prevalence of SLT use was generally low. The participants held a strong but inaccurate belief that SLT products were as harmful as smoked tobacco, which could result in less strenuous efforts to regulate smoked tobacco products. They felt that SLT products were more affordable, easier to conceal, and capable of being consumed more discreetly than cigarettes and noted that SLT use had negative consequences on Uganda’s economy. Interviewees believed that SLT products were illegally marketed and deceptively promoted as safe, food-like products. Participants thought that because of their appeal, their use was increasing among young people and women in Uganda. Therefore, there is a need for more research to explore the issues raised, including raising awareness of SLT, the absolute and relative harms compared with smoking, and exploring the addictiveness of SLT. This should help to strengthen enforcement of the comprehensive SLT ban in accordance with Uganda’s 2015 Tobacco Control Act to prevent the initiation of SLT use among non-smokers, especially women and children, whilst also ensuring a continuing focus on reducing smoking and combustible tobacco products.

### 5.1. Strength of the Study

This is the first study to provide an in-depth understanding of perceptions on SLT from enforcement stakeholders that have not previously been consulted.

### 5.2. Study Limitations

This being a qualitative study with a small sample size (*n* = 20), so generalizability of the findings to the whole country is a limitation. However, generalizability is not of so much priority in qualitative research (Valera et al., 2016). There were low levels of awareness of the law and SLT products, hence making it difficult for some participants to sufficiently discuss some aspects on the ban as well as their feelings about the products. Finally, the data collection stage coincided with the COVID-19 pandemic and the national response that necessitated that some interviews be conducted virtually. Consequently, unlike face-to-face sessions, the online discussions limited the extent of probing and length of the interviews.

## Data Availability

The data presented in this study are available on request from the corresponding author. The data are not publicly available due to privacy or ethical reasons.
